# Minimal information for chemosensitivity assays (MICHA): a next-generation pipeline to enable the FAIRification of drug screening experiments

**DOI:** 10.1093/bib/bbab350

**Published:** 2021-09-01

**Authors:** Ziaurrehman Tanoli, Jehad Aldahdooh, Farhan Alam, Yinyin Wang, Umair Seemab, Maddalena Fratelli, Petr Pavlis, Marian Hajduch, Florence Bietrix, Philip Gribbon, Andrea Zaliani, Matthew D Hall, Min Shen, Kyle Brimacombe, Evgeny Kulesskiy, Jani Saarela, Krister Wennerberg, Markus Vähä-Koskela, Jing Tang

**Affiliations:** Research Program in Systems Oncology, Faculty of medicine, University of Helsinki, Finland; Research Program in Systems Oncology, Faculty of medicine, University of Helsinki, Finland; Research Program in Systems Oncology, Faculty of medicine, University of Helsinki, Finland; Research Program in Systems Oncology, Faculty of medicine, University of Helsinki, Finland; Research Program in Systems Oncology, Faculty of medicine, University of Helsinki, Finland; Istituto di Ricerche Farmacologiche Mario Negri IRCCS, Italy; Institute of Molecular and Translational Medicine, Czech; Institute of Molecular and Translational Medicine, Czech; European Infrastructure for Translational Medicine, UK; Fraunhofer Institute for Molecular Biology and Applied Ecology, Germany; Fraunhofer Institute for Molecular Biology and Applied Ecology, Germany; National Center for Advancing Translational Sciences, USA; National Center for Advancing Translational Sciences, USA; National Center for Advancing Translational Sciences, USA; Institute for Molecular Medicine Finland, University of Helsinki, Finland; Institute for Molecular Medicine Finland, University of Helsinki, Finland; Biotech Research & Innovation Centre (BRIC), University of Copenhagen, Denmark; Institute for Molecular Medicine Finland, University of Helsinki, Finland; Research Program in Systems Oncology, Faculty of medicine, University of Helsinki, Finland

**Keywords:** drug discovery, drug sensitivity assays, data integration tools, FAIR research data

## Abstract

Chemosensitivity assays are commonly used for preclinical drug discovery and clinical trial optimization. However, data from independent assays are often discordant, largely attributed to uncharacterized variation in the experimental materials and protocols. We report here the launching of Minimal Information for Chemosensitivity Assays (MICHA), accessed via https://micha-protocol.org. Distinguished from existing efforts that are often lacking support from data integration tools, MICHA can automatically extract publicly available information to facilitate the assay annotation including: 1) compounds, 2) samples, 3) reagents and 4) data processing methods. For example, MICHA provides an integrative web server and database to obtain compound annotation including chemical structures, targets and disease indications. In addition, the annotation of cell line samples, assay protocols and literature references can be greatly eased by retrieving manually curated catalogues. Once the annotation is complete, MICHA can export a report that conforms to the FAIR principle (Findable, Accessible, Interoperable and Reusable) of drug screening studies. To consolidate the utility of MICHA, we provide *FAIRified* protocols from five major cancer drug screening studies as well as six recently conducted COVID-19 studies. With the MICHA web server and database, we envisage a wider adoption of a community-driven effort to improve the open access of drug sensitivity assays.

## Introduction

Drug sensitivity or chemosensitivity assay is an important tool to measure cellular response to drug perturbation, which has been increasingly used for preclinical drug discovery and clinical trial optimization. However, poor inter- and intralaboratory reproducibility has been reported when comparing batches that differ at assay conditions [1–3]. Central to improving the data reproducibility is the standardization of material and method descriptions, summarized as protocols, which should be sufficiently annotated and easily comparable. To make the assay protocols FAIR (Findable, Accessible, Interoperable and Reusable), a large variety of efforts to define the minimal information (MI) for specific assay types have been developed. In total, Minimum Information for Biological and Biomedical Investigations has reported 40 MI-based initiatives [4]. Among these, protocols for common omics assays include Minimal Information About Microarray Experiment, Minimum Information About a Next-generation Sequencing Experiment, Metabolomics Standards Initiative and Minimum Information About a Proteomics Experiment. For bioactivity assays in general, Minimum Information About Bioactive Entity (MIABE) has been widely used [5]. However, MIABE does not include specific guidelines for annotating drug sensitivity assays. Furthermore, like many other MI efforts, there is a lack of data integration tools to facilitate its implementation.

With an increasing number of drug sensitivity studies, efficient experimental annotation is critically needed to ensure the accessibility and reuse of the data. The solution we present here, MICHA (https://micha-protocol.org/), includes a guideline to annotate the MI for four major components of a drug sensitivity assay, including 1) compounds, 2) samples, 3) reagents and 4) data analysis. Furthermore, to make the annotation as efficient as possible, MICHA provides an integrative web tool that allows a user to retrieve the information about these assay components from public databases by standardized identifiers and ontologies. Without MICHA, a user would need to annotate a drug sensitivity experiment by retrieving multiple databases separately, which is often time-consuming and error prone. With the help of MICHA, we have catalogued the major drug sensitivity screening protocols in cancer and COVID-19 that may help users assess the FAIRness of existing experiments as well as inform the design of new experiments.

## Materials and methods

### Workflow

**Figure 1:**
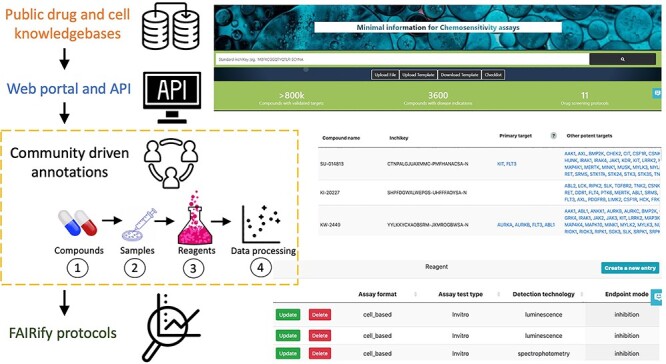
User interface and workflow of MICHA. Users start with compound annotation by uploading a list of compounds with names and standard InChiKeys. MICHA will return the pharmacological (drug targets, disease indications) and physiochemical properties of the compounds via an integrative web server and database, available under the ‘Compounds’ tab. Users then may click on Samples, Reagents or Data processing tabs to annotate their drug screening protocols. Auto suggestions are provided to avoid spelling mistakes or terminology conflicts. Finally, users can download the summary reports containing input data as well as integrated information provided by MICHA.

Using MICHA, users can upload their compounds, samples and experimental design information (Figure 1). To start, users need to upload the names and InChiKeys for the compounds, after which MICHA will automatically extract primary and secondary target information, physiochemical properties and disease indications. This information will help users annotate the mechanisms of action of the compounds. After obtaining the compound annotations, users may continue filling in the other experimental details, such as sample (cell lines or patient-derived samples) information and assay conditions. For cell lines, only the names of the cell lines are required, as the other information will be retrieved automatically from internal databases. For annotating assay protocols, we derive a consensus on the MI that is needed, including assay format, detection technology, end point mode of action, experimental medium, plate type, cell density, time for treatment, dilution fold, vehicle of compound, dispensation method and volume per well. These terms are defined in Supplementary File 1 as well as in the ‘Glossary’ tab at the MICHA website. Most of the terms are linked with the BioAssay ontology [6], which is commonly used for high-throughput chemical biology experiments [7]. Next, users are directed to a web form to report data processing information, including minimal and maximal concentrations of the compounds, publication references and drug response metric types such as IC_50_ or area under the dose–response curve (AUC). Finally, a tabular report can be generated according to the user’s input augmented with information retrieved from public resources (Supplementary File 2). In addition, MICHA provides a checklist of annotation items (Supplementary File 3). When preparing a manuscript, it is recommended to use the checklist to confirm the MICHA compliance, so that journals and reviewers can evaluate the FAIRness of the experiment more easily and more systematically. The Supplementary File 4 shows example template for FAIRified data that should be requested from the authors by the journals or reviewers.

### Data integration tools

Three types of datasets are retrievable via data integration tools in MICHA:

#### FAIRified protocols

A prime objective for MICHA is to provide a pipeline for the FAIRification of drug sensitivity assays, such that these established protocols can be well documented with enhanced visibility to the research community. To initiate such an effort, we have FAIRified drug screening protocols from major cancer studies including GDSC (345 compounds and 987 cell lines) [8], CCLE (24 compounds and 504 cell lines) [9] and CTRPv2 (203 compounds and 242 cell lines) [10]. Furthermore, we have provided drug sensitivity screening protocols extracted from six recent COVID-19 antiviral studies (5525 compounds and 2 cell lines) [11–16]. On the other hand, we have provided an example of protocols established at the research institution level (528 compounds and 4 cell lines utilized at the high-throughput Drug-Screening Unit at the Institute for Molecular Medicine Finland, University of Helsinki). These FAIRified protocols can be freely obtained at http://micha-protocol.org/protocols/. With more protocols annotated via MICHA, the drug discovery and translational medicine community shall be better informed on the variations on the experimental condition across different studies and institutions. Table 1 shows an overview of the FAIRified protocols by MICHA.

**Table 1:** FAIRified protocols by MICHA

Protocol name	Type	Comp-ounds	Cell lines	Detection technology	Dilution fold	Plate type	Min concentration (nM)	Max concentration (nM)	Metric	References
GDSC	Cancer	345	987	Fluorescence	2	384 and 96	0.03	4000 000	IC_50_	[10]
CCLE	Cancer	24	504	Luminescence	3.16	1536	2.5	8000	IC_50_, EC50	[9]
CTRPv2	Cancer	203	242	Luminescence	2	384	0.56	592,000	IC_50_	[8]
FIMM	Cancer	528	4	Fluorescence	10	384	0.0	1000 000	DSS	[17]
Mario Negri	Cancer	1	16	Spectrophotometry, luminescence	10	96	0.0	10 000	AUC	[18]
NCATS	COVID-19	5430	1	Luminescence	2–5	384	0.0	120 000	AC50	[15]
Ellinger *et al*.	COVID-19	103	1	Label free	3.33	384	20	20 000	IC_50_, CC50	[16]
Gordon *et al*.	COVID-19	73	1	Spectrophotometry		96	1	10 000	IC_50_	[12]
Jeon *et al*.	COVID-19	43	1	Microscopy	2	384	50	50 000	IC_50_	[14]
Touret *et al*.	COVID-19	83	1	qPCR	2	96	600	40 000	EC_50_	[13]
Weston *et al*.	COVID-19	43	1	Luminescence	2	96	50	5000	IC_50_	[11]

**Figure 2:**
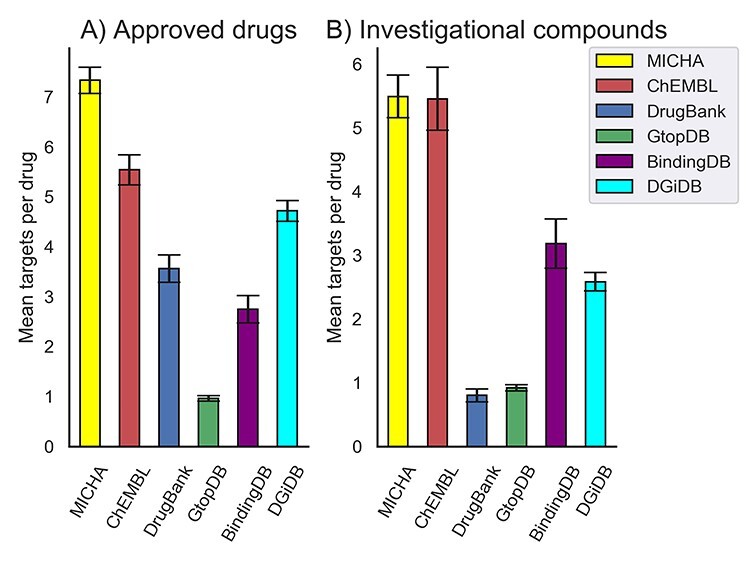
Average number of targets for compounds in multiple databases. (**A**) Approved drugs. (**B**) Investigational compounds.

#### Compound target profiles

Compound–target profiles are integrated from the most comprehensive drug–target databases including DrugTargetCommons (DTC) [19] [20], BindingDB [21], ChEMBL [22], GtopDB [23], DGiDB [24] and DrugBank [25]. The first four databases (DTC, BindingDB, ChEMBL and GtopDB) contain quantitative bioactivity data, whereas DGiDB and DrugBank contain unary drug–target information. We have focused on the primary and secondary targets of a compound, defined as those displaying binding affinities ≤ 1000 nM from the bioactivity databases, or those that are recorded in the unary databases. We have integrated drug targets for 277K chemicals from DTC, 513K from ChEMBL, 258K from Binding DB, 4.8K from GtopDB, 7.6K from DGiDB and 6.8K from DrugBank. Furthermore, we have merged overlapping targets across these databases to avoid duplications, resulting in high-quality target profiles for >800K chemicals. Such a data integration provides one of the most comprehensive compounds collection along with their potent primary and secondary targets.

#### Compound properties, cell line and assay information

Compound physiochemical properties and structures for 1.9 million compounds are obtained from the ChEMBL database. Furthermore, we have integrated disease indications and clinical phase information for 3600 clinical drugs from the DTC database. This information together with the drug–target profiles will be retrieved for user-uploaded compound list. When users annotate the cell lines, the majority of cell line information can be retrieved automatically from Cellosaurus [26], which is a comprehensive knowledge database on cell lines. For assay annotation, commonly used techniques will be provided for users to choose from to ease the burden of manual editing.

## Added values by MICHA

### Comprehensive compound–target profiles

For annotating the mechanisms of action of compounds, MICHA integrates compound–target profiles from various databases, ranging from quantitative bioactivity values to unary drug–target hits. For instance, DrugBank, GtopDB and DGiDB are mainly focused on approved compounds with putative target information, whereas ChEMBL, BindingDB and DTC include bioactivity values for more versatile investigational and preclinical chemicals. In MICHA, we have improved target coverage across the druggable genome by integrating nonoverlapping data points from the latest releases of these databases. As shown in Figure 2, the average number of targets for 2993 approved drugs (and salts) in MICHA is 7.33, as compared with that from ChEMBL (5.5), DGiDB (4.71), DrugBank (3.56), BindingDB (2.74) and GtopDB (0.96). Similarly, for 1992 investigational compounds (defined as those in clinical trials), the average number of targets per chemical is higher in MICHA as compared with other databases. Secondly, MICHA provides efficient Application Programming Interface (API) for retrieving comprehensive target profiles, available at: https://api.micha-protocol.org (Supplementary File 5). We believe that the API for compound–target information will further boost the usability of MICHA by programmatically integrating compound–target profiles with other related tools and shall open new applications for drug discovery researchers for training their compound–target machine learning models [27–29] as well as providing more insights on the network modeling of mechanisms of action [30, 31].

### Systematic comparison of drug sensitivity screening protocols

We have FAIRified the screening protocols for three major cancer drug studies including CCLE, GDSC and CTRPv2 (Table 1). FAIRification of these protocols is performed using MI-based information as mentioned in checklist available at the home page of MICHA (as well as in Supplementary File 3). These drug screening studies share similar objectives of linking genetic features of cancer cell lines to small-molecule sensitivity to accelerate drug discovery. Note that MICHA focuses on the annotation of drug screening protocols while the actual data points are available in their corresponding databases. Here we report the comparison of the major components in the assay protocols (Table 2).

**Table 2:** Comparison between protocols of CCLE, GDSC and CTRPv2

	CCLE	GDSC	CTRPv2
Plate types	1536	384, 96	384-Opaque white
Experimental mediums	NA	DMEM, RPMI	ALPHAMEM, DMEM, DMEMF, EMEM, HAMSF, IMDM, L15, McCoys5A, MCDB, MEM, RPMI, Waymouth, WilliamsE
Detection technology	Luminescence	Fluorescence	Luminescence
Cell density	250	NA	500, 1000, 2000, 3000, 5000, 10 000
Treatment time (h)	72–84	72	72
Analysis metrics	IC_50_, EC_50_	IC_50_	IC_50_
Compounds	24	345	203
Number of cell lines	504	987	242

NA indicates that the information is unavailable.

Both GDSC and CTRPv2 used common experimental plate type i.e. 384 wells, whereas CCLE compounds were tested on 1536 well plates. In GDSC, two different experimental mediums including DMEM and RPMI were tested for the 987 cancer cell lines, whereas the CTRPv2 cell lines were tested for many more different mediums as listed in Table 2. In contrast, we could not find experimental medium information for CCLE. On the other hand, both CCLE and CTRPv2 have used Cell-Titer-Glo (Promega), a luminescence-based assay to measure the levels of ATP as a surrogate to cell viability, whereas GDSC has used based nucleic acid staining syto60 (Invitrogen) for adherent cells and resazurin (Sigma) for suspension cells. All the three screening studies have used at least 72 h of treatment, after which the IC_50_ or EC_50_ concentrations were determined from the dose–response curves.

**Figure 3:**
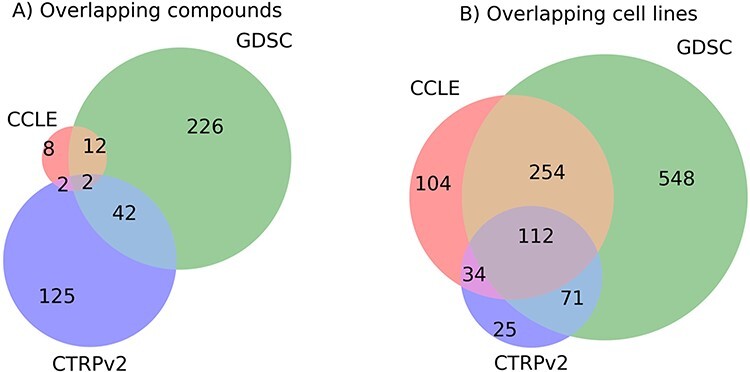
Overlapping data between CCLE, GDSC and CTRPv2 database. (**A**) Overlapping compounds. (**B**) Overlapping cell lines.

Figure 3 shows the overlapping chemicals and cell lines tested across CCLE, GDSC and CTRPv2 studies, after excluding those chemicals for which proper chemical names or identifiers were missing to assure high-quality data in MICHA. Only two chemicals are shared across the three studies including selumetinib and tanespimycin (Figure 3**A**). Selumetinib (AZD6244) is a MEK (kinase) inhibitor used for treating neurofibromatosis type I in children [32], whereas tanespimycin is a Hsp90 inhibitor [33] that has been studied for the treatment of leukemia or solid tumors, especially kidney tumors. In contrast, more overlap was found for the cell lines, with 112 cell lines in common across the three studies (Figure 3**B**).

MICHA has not only FAIRified cancer-related drug screening studies but also annotated six recent studies on COVID-19, a virus that causes ongoing pandemic with limited drug treatment options. From these studies, we have identified 5525 chemicals tested across two cell lines including Vero E6 and Caco-2. The annotations of these compounds, cell lines as well as the experimental information and data analysis methods can be easily retrieved at http://micha-protocol.org/covid19.

**Figure 4:**
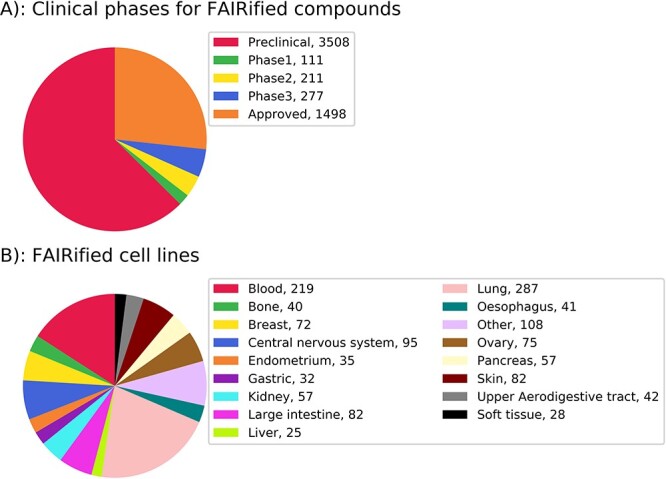
(**A**) Clinical phase of FAIRified compounds. (**B**) Tissue types for FAIRified cell lines.

In total, Figure 4**A** shows the clinical phases of the compounds FAIRified by MICHA, whereas Figure 4**B** shows the distribution of FAIRified cell lines from different tissue types. These statistics show a broad coverage of cell lines and compounds. We believe that with the FAIRification of more protocols, MICHA has the potential to become a standard workflow for annotating and cataloguing chemosensitivity experiments.

With the help of MICHA platform, all these COVID-19- and cancer-related drug screening protocols are freely accessible to the users (Findable and Accessible). Moreover, these protocols can be accessed programmatically using MICHA API, which makes it possible for *in silico* models to programmatically access MICHA to obtain compound information such as protein targets and physiochemical properties and use this information for novel drug–target predictions (Interoperable). The MICHA drug screening protocols can be considered as a reference for the experimental design of future drug screening studies as well as serve as a source of information to evaluate the experimental reproducibility (Reusable). MICHA is also indexed at https://fairsharing.org/ to be accepted as a potential tool for chemosensitivity data FAIRification.

## Conclusion

Chemosensitivity assay screening has been increasingly utilized for preclinical drug discovery and clinical trial optimization. However, chemosensitivity assays often lack sufficient annotation to make the data FAIR, which has become a limiting factor for supporting its clinical translation. To improve the assay annotation, web portals that facilitate information retrieval from different assay components are critically needed. To address this issue, we have recently launched MICHA as a web server for the annotation of chemosensitivity screens that covers critical information including 1) compounds 2) samples 3) reagent protocols and 4) data processing methods.

The novelty of MICHA is 2-folds. First, it provides a protocol for defining MI for annotating drug sensitivity assays. Second, it provides software tools to implement such a protocol. To enable an effective data annotation pipeline, comprehensive compound–target profiles are deposited to the MICHA database for more than 800K compounds. These high-quality pharmacological data shall help improve the annotation on the mechanisms of action not only for approved drugs but also for investigational and preclinical compounds. Furthermore, the target profiles at the druggable genome scale provide more information on the polypharmacological effects, which might lead to new opportunities for drug repositioning [34]. To facilitate the data retrieval, the API in MICHA is highly optimized such that it can return target profiles for hundreds of compounds within seconds.

With the MICHA web portal, we have FAIRified major drug sensitivity screening protocols from five cancer studies and six recent COVID-19 studies, serving as the first instances of the catalogue. Comparing these deeply curated assay protocols should allow a more systematic analysis of data reproducibility. With the FAIR-compliant data resources and tools to deliver content standards and ontology services, MICHA will ensure the characterization of critical assay components, allow the FAIRification and cataloguing of drug sensitivity studies and support the downstream analysis toward clinical translation. We invite the drug discovery community to use MICHA for annotating their drug sensitivity assays to improve the knowledge sharing, which shall ultimately lead to a bigger impact in translational medicine.

**Key Points**

- We proposed a novel workflow called MICHA (https://micha-protocol.org) for the FAIRification of drug sensitivity screening protocols.
- MICHA provides an integrated platform to obtain drug screening assay annotations, drug–target profiles and other pharmacological information in an easy and fast manner.
- MICHA FAIRified drug screening protocols related to cancer and COVID-19, which are made comparable and informative for designing new experiments.

## Data availability statement

FAIRified protocols by MICHA are freely accessible using MICHA API as well as using a standalone file at https://micha-protocol.org/download/index. Compound–target profiles can be retrieved by the MICHA annotation pipeline or programmatically using the API. Furthermore, compound–target profiles can also be downloaded as a standalone file at https://micha-protocol.org/download/index.

## Supplementary Material

Supplementary_File_1_bbab350

Supplementary_File_2_bbab350

Supplementary_File_3_bbab350

Supplementary_File_4_bbab350

Supplementary_File_5_bbab350

## Notes

**Ziaurrehman Tanoli** is senior researcher at University of Helsinki. His research is mostly focused on computational drug repurposing. He is also developing bioinformatics tools for drug–target interactions.

**Jehad Aldahdooh** is a PhD student at University of Helsinki. He is developing text mining applications for drug–target interactions.

**Farhan Alam** is PhD student at University of Helsinki.

**Yinyin Wang** is a PhD student at University of Helsinki. She is developing methods for network pharmacology modeling for herb medicine.

**Umair Seemab** is PhD student at University of Helsinki. He is working on NGS and single-cell sequencing.

**Maddalena Fratelli** is head of Phamacogenomics Unit at Mario Negri Institute for Pharmacological Research. She is working on genomic and transcriptomic systems for the study of drug action and resistance.

**Petr Pavlis** is senior developer at Institute of Molecular and Translational Medicine. He is developing bioinformatics applications.

**Marian Hajduch** is founding director of the Institute of Molecular and Translational Medicine. His research interests are in molecular and translational medicine.

**Florence Bietrix** is the head of operations at EATRIS. Her research interests are to develop new therapeutic targets for atherosclerosis and nonalcoholic steatohepatitis.

**Philip Gribbon** is head of Discovery Research at Fraunhofer Institute for Translational Medicine and Pharmacology.

**Andrea Zaliani** is senior bioinformatics scientist at Fraunhofer Institute for Translational Medicine and Pharmacology. He has expertise in pharmaceutical research and development.

**Matthew D. Hall** is biology group leader at National Center for Advancing Translational Sciences. He optimizes both biochemical and cell-based assays for automated, small molecule, high-throughput screening in collaboration with NIH.

**Min Shen** has extensive experience in cheminformatics and computational chemistry through her work at NCATS.

**Kyle Brimacombe** is biochemist at National Center for Advancing Translational Sciences.

**Evgeny Kulesskiy** is senior researcher at Institute for Molecular Medicine Finland. He is working on drug sensitivity and resistance testing.

**Jani Saarela** is head of High Throughput Unit at Institute for Molecular Medicine Finland. He is working on drug sensitivity and resistance testing.

**Krister Wennerberg** is professor at Biotech Research & Innovation Centre. He is doing research on chemical systems biology applications.

**Markus Vähä-Koskela** is senior researcher at Institute for Molecular Medicine Finland. He is doing research in areas such as: onco-immunology, immunotherapy and translational cancer medicine.

**Jing Tang** is assistant professor at University of Helsinki. He is working on mathematical, statistical and informatics tools to tackle biomedical questions.
