# Supplementary material for: Minimal information for chemosensitivity assays (MICHA): a next-generation pipeline to enable the FAIRification of drug screening experiments
Source: Brief Bioinform. 2021 Sep 1;23(1):bbab350. doi: 10.1093/bib/bbab350 (PMC8769689; doi:10.1093/bib/bbab350)
Supplement: Supplementary_File_1_bbab350 [file supplementary_file_1_bbab350.pdf]

Glossary for terms in MICHA (https://micha-protocol.org/glossary/index)

Following is the glossary for the terms used in MICHA drug screening protocol. Some of the fields are associated with Bioassay ontology (BAO) terms using IDs e.g., ***BAO_0000551, BAO_0000238.***

1) **Samples (cell lines)**

   If data is focusing on cell lines, users have to enter following cell line information. Highlighted fields can be automatically extracted by MICHA and users do not need to enter.

   - ***Name:*** *Name of the cell line (e.g. MOLM-13, DU4475)*
   - ***Cellosaurus ID:*** *ID for cell line*
   - ***Cell type:*** *It shows the tissue type for cell line e.g. acute myeloid leukemia or breast cancer.*
   - ***Cell line organism (BAO_0000551):*** *Cell line specie e.g. Homo sapiens.*
   - ***Cell line modifications (BAO_0000238):*** *Modification to actual cell line*

2) **Samples (patient)**

   If data is focusing on patients, users have to enter following patient details information. Patient ID is optional.

   - ***Patient ID:*** *Patient id, this field is optional.*
   - ***Patient age:*** *Age of the patient*
   - ***Patient sex:*** *Male or female*
   - ***Patient Diagnosis:*** *Disease name e.g. acute myeloid leukemia or breast cancer*
   - ***Patient sample material:*** *For example, bone marrow, peripheral blood*
   - ***Patient sample date:*** *Date at which sample was collected (MM/DD/YYYY)*
   - ***Patient date of evaluation:*** *Date at which patient sample was evaluated (MM/DD/YYYY)*

3) **Compound**

   Comprehensive information on screened compounds. Most of this information is automatically extracted from public resources such as ChEMBL(https://www.ebi.ac.uk/chembl/), DrugComb (https://drugcomb.fimm.fi/) and ClinicalTrials(https://www.clinicaltrials.gov/). Users just need to upload Compound names and standard InChiKeys. For instance, standard InChiKey for imatinib is: XDXDZDZNSLXDNA-TZNDIEGXSA-N.

- **Clinical Phase:** This is the max clinical phase for compound that has been clinically tested. This is automatically extracted by MICHA using ChEMBL API.
- **Links to clinical studies:** Links to the ongoing or completed clinical studies.
- **Cross referencing drug databases:** Provide hyperlinks to other compound databases such as: Pubchem, ChEMBL, DTC, BindingDB, Drugbank, Zinc, emolecules, atlas, gtopDB, Chebi, PharmGKB, sure ChEMBL, Lincs.
- **Molecule type:** For example, Small molecular, antibody
- **Physiochemical properties:** Nearly 20 physiochemical properties can be automatically extracted by MICHA such as: ALOGP, number hydrogen bond acceptors (HBA) and number of aromatic rings etc.
- **Disease indication:** Disease indications which are currently tested or under trial. This can be automatically extracted based on InChiKey.
- **Primary target:** Primary protein targets for the compounds can be automatically extracted, if available in public databases (ChEMBL, Drug Target Commons, PubChem and DrugBank).
- **Off targets:** Potent protein targets other than primary targets can also be automatically extracted from public databases such as: (ChEMBL, Drug Target Commons, PubChem and DrugBank).
- **Concentration range:** Users must have to provide minimum and maximum concentration used for their experiments in nM.
- **Dilution steps:** For example, ten-fold, half-log
- **Vehicle of compound:** For example, DMSO/H2O

4) Experiments

Following is the description about experimental terms used in MICHA.

- **Medium (BAO_0000596)**: Examples for medium are: RPMI1640, 10% FBS, pen/strep, glutamine
- **Plate type (BAO_0000508):** Examples for plate types are: Corning 384-well # 3764
- **Surviving cells (%):** Percentage of surviving cells.
- **Volume/well:** This information must be provided in ul.
- **Time of treatment:** Such as 72 hours

- **Cell density (BAO_0000572):** Number of cells per well e.g 3000.
- **Method of dispensing:** Such as Biotek Multiflo FX with RAD cassette (1 channel)
- **Measurement type:** It shows the experimental data metric such as EC50, IC50, DSS etc
- **Measurement value:** This must be entered into Nano molar units.

5) Assay formats (BAO_0000019)

An assay format is a conceptualization of assays based on the biological and/or chemical features of the experimental system. For example, assay formats include 1) biochemical, assays with purified protein, 2) cell-based, assays in whole cells, 3) cell-free, assays in cell derivatives, 4) organism-based, assays performed in an organism, 5) physiochemical, assays which measure physical or chemical properties, and 6) tissue-based, assays using tissue derived from a living organism. **This information must be selected from drop down list in MICHA excel template.**

- ***Biochemical (BAO_0000217)****: A biochemical assay format is an in vitro format used to measure the activity of a biological macromolecule, e.g. a purified protein or nucleic acid. It is most often a homogeneous assay, but can be heterogeneous if a solid phase, such as beads, is used to immobilize the macromolecule.*
- ***Cell-based (BAO_0000219):*** *A cell-based assay format involves the use of living eukaryotic cells and is a heterogeneous assay.*
- ***Cell-free (BAO_0000366):*** *A cell-free assay format originates from cells, but does not use intact, live cells. This format is distinct from biochemical assays. It is most often a homogeneous assay, but can be heterogeneous if a solid phase, such as beads, is used to immobilize the components.*
- ***Organism-based (BAO_0000218):*** *An organism-based assay format involves the use of a living organism and is a heterogeneous assay.*
- ***Physiochemical (BAO_0000684, BAO_0010009):*** *A physiochemical assay format involves the measurement of physical and chemical properties of perturbagens, namely aqueous solubility, octanol/water partition, or cell permeability models e.g. parallel artificial membrane permeability assay (PAMPA).*
- ***Tissue based (BAO_0000221):*** *A tissue-based assay format involves the use of a tissue derived from a living organism and is a heterogeneous assay type.*

6) **Detection technologies (BAO_0000035):** A detection technology is the physical method or technique readout used to measure an effect caused by a perturbagen in an assay environment.

- **Fluorescence (BAO_0000046):** Fluorescence detection methods use the principles of fluorescence, whereby incident light excites a fluorophore that then emits light at lower energy and higher wavelength, typically in the visible portion of the UV-Visible spectrum.
- **Fluorescence polarization (BAO_0000003):** Fluorescence polarization (FP) measurements are based on the assessment of size-dependent rotational motions of species and used to measure binding interactions.
- **Alpha Screen (BAO_0000130):** Amplified Luminescent Proximity Homogeneous Assay Screen is a subtype of fluorescence detection technologies.
- **Time Resolved Fluorescence (BAO_0000004):** Time Resolved Fluorescence (TRF) is a subtype of fluorescence detection technologies. One commercial name is DELFIA.
- **Time -Resolved Fluorescence Energy Transfer (BAO_0000004):** Time-Resolved Fluorescence Energy Transfer (TR-FRET) is a subtype of fluorescence detection technologies. Commercial names are LANCE and HTRF.
- **Label-free technology (BAO_0000427):** Label-free detection technologies measure binding interactions and cell-based reactions in the absence of conventional labels, e.g., fluorescent probes. Advantages include the ability to measure a) functional activity without modifying the binding partners with labels, b) binding interactions independent of functional activity, and c) cell-based assays without the need to engineer cell-lines to over-express given targets, such as GPCRs.
- **Luminescence (BAO_0000045):** Luminescence detection technologies make use of light emission that occurs from an electronically excited state reached by a physical, mechanical, or chemical mechanism.
- **Microscopy (BAO_0000452):** Microscopic detection technologies use microscopes to see objects that cannot be seen with an unaided eye.
- **Quantitative PCR (BAO_0002089):** Quantitative PCR, sometimes referred to as RT-PCR, detection technologies use DNA labeled protein in a binding assay and are

detected by quantifying the amount of DNA by PCR. A commercial assay called KINOMEscan was specifically developed for screening purposes.

- **Radiometry (BAO_0000657):** Radiometry detection technologies use radioactive tracers. Examples of assays that use radiometry are filter assays and Scintillation Proximity Assay (SPA).
- **Spectrophotometry (BAO_0000049):** Spectrophotometry detection technologies measure the amount of light that a sample absorbs. A spectrophotometer operates by passing a beam of light through a sample and measuring the intensity of light reaching a detector.
- **Thermal shift (BAO_0000058):** Thermal shift detection technologies detect temperature shifts using a fluorescent dye that is sensitive to a protein environment. Upon heating, a protein unfolds and loses native conformation. Binding of a small molecule can often stabilize the protein conformation, resulting in a higher unfolding temperature.
