# Supplementary material for: Minimal information for chemosensitivity assays (MICHA): a next-generation pipeline to enable the FAIRification of drug screening experiments
Source: Brief Bioinform. 2021 Sep 1;23(1):bbab350. doi: 10.1093/bib/bbab350 (PMC8769689; doi:10.1093/bib/bbab350)
Supplement: Supplementary_File_3_bbab350 [file supplementary_file_3_bbab350.pdf]

MICHA CHECKLIST

Following is the checklist of minimum information required by MICHA guidelines. The terminologies in blue text are suggested as mandatory to be provided by the users.

1: COMPOUND INFORMATION

- Standard InChiKey
- Name
- Smiles

2: SAMPLE INFORMATION

Cell line

- Name
- Provenance
- ID
- Passage number
- Modifications

OR

Sample

- ID
- Age
- Sex
- Diagnosis
- Sampling material
- Date of sampling
- Date of evolution

3: REAGENTS

- Dilution fold (e.g. 2, 5, 10)
- Vehicle (e.g. DMSO)
- Experimental medium (e.g. DMEM)
- Plate type (e.g. 384-well)
- Assay format (e.g. cell based, organsim based, cell free, biochemical)
- Detection technology (e.g. qPCR, luminescence, label free, spectrophotometry, microscopy, Fluorescence)
- Cell density at plating (cells/well)
- Method of dispensing (e.g. Biotek Multiflo FX with RAD cassette)
- Volume per well (e.g. 20uL)
- Time of treatment (e.g. 120 hours)

4: DATA PROCESSING METHOD

- Minimum tested concentration (in nM)
- Maximum tested concentration (in nM)
- Analysis metric (e.g. AC50, DSS)
- Analysis reference (e.g. PubMed ID, or weblink)
