# Supplementary material for: Minimal information for chemosensitivity assays (MICHA): a next-generation pipeline to enable the FAIRification of drug screening experiments
Source: Brief Bioinform. 2021 Sep 1;23(1):bbab350. doi: 10.1093/bib/bbab350 (PMC8769689; doi:10.1093/bib/bbab350)
Supplement: Supplementary_File_5_bbab350 [file supplementary_file_5_bbab350.pdf]

APPLICATION PROGRAMMING INTERFACE (API) FOR MICHA

MICHA provides API to give programmatic access over FAIRified drug screening protocols, target data profiles and disease indications for 0.8M compounds.

API for FAIRified drug screening protocols

So far, our annotation team has FAIRified 11 protocols including 5 cancer protocols and 6 covid19 protocols. But we will FAIRify more protocols in future. Altogether, MICHA FAIRified 5,605 compounds and 1,377 cell lines. This information can be programmatically extracted and integrated with other public tools using API under FAIR principals. Users can extract data using various options and apply different filters to get specific results. Following are some of the examples on how to extract FAIRified protocols.

1) ***FAIRified drug screening data using compound names***

Users can extract information from any of the FAIRified protocols using compound names. Compound names are case insensitive. Following link returns FAIRified drug screening data for gefitinib, which is an approved EGFR inhibitor.

https://api.micha-protocol.org/annotations/?compound_name=Gefitinib

2) ***FAIRified drug screening data using standard InChiKeys***

Though approved compounds have proper names but there exist millions of preclinical or investigational compounds that are not yet assigned with proper names. These compounds are usually referenced using standard identifiers such as smiles or InChiKeys. Therefore, MICHA also support querying FAIRified compounds using standard InChiKeys. Following link returns FAIRified drug screening data for gefitinib using its standard InChiKey.

https://api.micha-protocol.org/annotations/XGALLCVXEZPNRQ-UHFFFAOYSA-N

3) ***FAIRified drug screening data using cell line names***

Users can also query FAIRified cell lines using cell line name as shown in the following link:

https://api.micha-protocol.org/annotations/?cell_line=MOLM-13

Furthermore, users can also apply multiple filters separated by '&' operator. For instance, FAIRified compound across a particular cell line can be obtained as.

https://api.micha-protocol.org/annotations/?cell_line=MOLM-13&compound_name=gefitinib

4) ***FAIRified drug screening data using thresholds on activity values***

Other than compound or cell line information users can also query FAIRified annotations using thresholds on minimum or maximum concentrations. For example, following link can return

all FAIRified compounds and cell lines with concentration: 1-1000 nM. We adapted nM as standard activity units in MICHA.

https://api.micha-protocol.org/annotations/?min_concentration=>1&max_concentration=<1000

5) FAIRified drug screening data using activity metric

There are several activity metrics used in different protocols such as IC50, EC50, DS and AC50. Users can also filter data using various metrics. For example, following link returns 'AC50' data between concentration: 1-1000 nM.

https://api.micha-protocol.org/annotations/?min_concentration=>1&max_concentration=<1000&analysis_metric=IC50

6) FAIRified drug screening data using detection technology

Following are the links to get FAIRified drug screening annotations for different detection technologies.

https://api.micha-protocol.org/annotations/?detection_tech=spectrophotometry

https://api.micha-protocol.org/annotations/?detection_tech=Fluorescence

https://api.micha-protocol.org/annotations/?detection_tech=qPCR

https://api.micha-protocol.org/annotations/?detection_tech=Microscopy

https://api.micha-protocol.org/annotations/?detection_tech=Luminescence

7) FAIRified drug screening data using other search queries

Other than aforementioned search queries, users can also query FAIRified drug screening data using various other options such as cell density, plate types, volume per well, vehicle etc. API links for these searches are shown below:

https://api.micha-protocol.org/annotations/?cell_density=250

https://api.micha-protocol.org/annotations/?plate_type=1536

https://api.micha-protocol.org/annotations/?volume_per_well=5

https://api.micha-protocol.org/annotations/?volume_per_well=DMSO

API for compound target profiles and disease indications

There are potent target profiles for ~0.8M compounds in MICHA. These target profiles can be extracted or linked with external tools using FAIR principals. Furthermore MICHA also

provides tested disease indications for the input compounds as well as physiochemical properties

1) ***Get target profile and disease indications for one compound***

Following link returns primary and other potent targets for gefitinib as well as disease indications that are currently under trials at: https://clinicaltrials.gov/.

https://api.micha-protocol.org/get_targets/XGALLCVXEZPNRQ-UHFFFAOYSA-N

2) ***Get target profiles for multiple compounds***

Users can also query more than 1 compound in one request. Micha allows target profiles for maximum of 500 compounds in one API request. However, users can fetch thousands of compounds using loops in the code. For querying multiple compounds users need to enter standard InChiKeys separated by comma. Following example returns compound target profiles as well as disease indications for three compounds (gefitinib, afatinib and apatinib)

https://api.micha-protocol.org/get_targets/XGALLCVXEZPNRQ-UHFFFAOYSA-N,ULXXDDBFHOBEHA-CWDCEQMOSA-N,FYJROXRIVQPKRY-UHFFFAOYSA-N
